# Natural Deep Eutectic Solvents as Green Alternatives for Extracting Bioactive Compounds from *Sideritis* Taxa with Potential Cosmetic Applications

**DOI:** 10.3390/antiox14010068

**Published:** 2025-01-09

**Authors:** Lamprini Zissi, Virginia D. Dimaki, Vassiliki S. Birba, Vassiliki C. Galani, Vassiliki Magafa, Sophia Hatziantoniou, Fotini N. Lamari

**Affiliations:** Department of Pharmacy, School of Health Sciences, University of Patras, 26504 Patras, Greece; zisilina95@hotmail.com (L.Z.); virnadimaki@upatras.gr (V.D.D.); up1060365@ac.upatras.gr (V.S.B.); galan.vasiliki@gmail.com (V.C.G.); magafa@upatras.gr (V.M.); sohatzi@upatras.gr (S.H.)

**Keywords:** *Sideritis*, antioxidant, green extraction, deep eutectic solvents, betaine, ironwort

## Abstract

This study investigated the potential of natural deep eutectic solvents (NADESs) for extracting bioactive compounds from the aerial parts of two mountain tea taxa, *Sideritis clandestina* ssp. *peloponnesiaca* (Boiss. & Heldr.) Baden and *Sideritis raeseri* Boiss. & Heldr. ssp. *raeseri*. Five NADEs, composed of betaine, glycerol, glucose, urea, citric acid, and sucrose, were evaluated for their extraction efficiency compared to conventional solvents (water and 70% ethanol). The total phenolic content (TPC) and antioxidant activity (FRAP and DPPH assays) were determined. Results showed that water was not a good extraction solvent. Despite the great solvent-dependent differences, most NADEs, particularly the betaine–glycerol–glucose mixture (BGG4), exhibited comparable or even superior extraction efficiency and antioxidant activity compared to 70% ethanol. The secondary metabolites in the BGG4 and 70% ethanol extracts were determined with HPLC-MS. The BGG4 extracts of both *Sideritis* taxa had a rich phenolic profile, with the major ingredients being chlorogenic acid, verbascoside, and non-, mono- and di-acetylated allosyl hypolaetin glycosides. Although distinct quantitative differences in their composition compared to the respective 70% ethanol extracts, and between them were noted, overall, the content of secondary metabolites in both *S. raeseri* extracts was lower than that of the *S. clandestina* extracts. These findings suggest that NADEs, particularly BGG4, are promising green solvents for extracting bioactive compounds from *Sideritis* taxa, paving the way for potential applications in the development of natural and sustainable cosmetic products.

## 1. Introduction

*Sideritis* herbs, commonly known as mountain tea or shepherd’s tea, belong to the Lamiaceae family and are widely consumed as beverages and used in traditional medicine. This genus, which encompasses around 150 species, is native to the Mediterranean, Central Europe, and temperate Asia. The plants are typically annual or perennial herbs, known for their resilience to drought and low temperatures. They thrive in steep, rocky terrains at altitudes exceeding 1000 m.

In Greece, 16 native species and subspecies of *Sideritis* thrive, belonging to the Empedoclia group [1]. *Sideritis clandestina* Bory & Chaub. (also known by its synonyms *Sideritis theezans* Boiss. & Heldr., *Sideritis cretica* Sibth & Sm., and *Sideritis syriaca* Bory & Chaub.) is endemic to the mountains of the Peloponnese. This species has two subspecies: ssp. *clandestina* and ssp. *peloponnesiaca*, with the latter prospering in the mountains of Mainalo, Mavrovouni, Oligyrtos, Kyllini, Helmos, Dourdouvana, and Erymanthos. *Sideritis raeseri* Boiss & Heldr. is native to the southern and western Balkan Peninsula, with two subspecies present in Greece: ssp. *attica* and ssp. *raeseri* [synonym: *Sideritis sicula* Ucria ssp. *raeseri* (Boiss. & Heldr.)]. The latter subspecies naturally grows in the mountains of Central Greece, including the Northern and Southern Pindos range, Eastern Central Greece, and North Central Greece, as well as across the mountains of the Western Balkans.

The European Medicines Agency (EMA) has approved *Sideritis* as a traditional herbal medicinal product for the relief of cough associated with colds and for mild stomach and bowel discomfort (EMA/HMPC/39453/2015). Preclinical studies have shown that *Sideritis* extracts possess anti-inflammatory, antimicrobial, and antioxidant properties, making them promising candidates for use in skincare products [2,3,4,5,6]. *Sideritis euboea* Heldr. extracts have anti-hyaluronidase activity [7]. In an evaluation of 440 plant taxa from Greece concerning their antioxidant, anti-melanogenic properties and their effect on key enzymes and processes of skin aging in vitro, *Sideritis scardica* Griseb. extracts had notable photoprotective and anti-aging activity [8]. In particular, *Sideritis raeseri* Boiss. & Heldr. ssp. *raeseri* (SR) hydroethanolic extract and its fractions have photoprotective, antioxidant, anti-tyrosinase, and antimicrobial activity against *Staphylococcus aureus*, *Staphylococcus epidermidis*, and *Pseudomonas aeruginosa* [9]. Furthermore, other *Sideritis* taxa extracts have been used for the successful green synthesis of silver nanoparticles with significant tyrosinase inhibitory activity [10]. Altogether, the results suggest that *Sideritis* extracts are valuable ingredients for skin-care cosmetic and topical pharmaceutical products.

*Sideritis* species are aromatic, with a low percentage of essential oil, and are rich in diterpenes. They also contain iridoids, phenolic acids, phenylethanoid glycosides, and unusual acetylated and non-acetylated allosyl flavone glycosides and their methyl ethers as their predominant constituents [6,11,12]. In our previous study, we isolated four iridoid glycosides (monomelittoside, melittoside, ajugoside, and 7-*O*-acetyl-8-epiloganic acid), two phenolic acid (vanillic and salicylic acid) glycosides, and three caffeoyl esters and glycosides (chlorogenic acid, verbascoside, and isoverbascoside) from *Sideritis clandestina* ssp. *peloponnesiaca* (Boiss. & Heldr.) Baden (SC) [13]. We also developed analytical methodologies to determine the major secondary metabolites in *Sideritis* taxa and revealed distinct phytochemical differences between SC and SR [13].

Conventional organic solvents such as ethanol, methanol, and acetone are commonly used to extract bioactive polar compounds. However, in the search for more sustainable green alternatives, natural deep eutectic solvents (NADESs) are being explored. In nature, common plant metabolites—such as sugars, organic acids, amino acids, and choline derivatives—can form eutectic mixtures that solubilize, store, and transport non-water-soluble metabolites [14]. These mixtures have a melting point significantly lower than those of the individual components. NADESs are non-flammable, non-toxic, and easy to prepare. Moreover, their ability to solubilize both polar and non-polar compounds, along with their low cost, safety, and the fact that many NADES ingredients are edible, makes them promising green solvents for a variety of industrial applications and a promising area for further research [14].

Researchers have investigated the use of NADESs as extraction agents for secondary metabolites such as polyphenols [15,16], ginsenosides [17], anthocyanins [18,19], and catechins [20]. In all studies, NADESs demonstrated higher extraction yields compared to conventional solvents. A 2017 study evaluated eleven NADESs for their potential in extracting, storing, and introducing natural products into cosmetic formulations [21]. The results were promising, showing that NADESs provided higher catechin extraction yields from tea than methanolic, ethanolic, and aqueous solvents. Additionally, NADESs better preserved catechins during storage [21]. Since then, many NADESs have been evaluated for the extraction efficacy of plant sources and their use in cosmetic products, since the NADES ingredients also have beneficial properties, are natural, and stabilize the mixtures of extracted compounds without preservatives [22]. To date, the only NADESs used for extraction of a *Sideritis* species (*S. scardica*) include choline chloride: glycerol (1:2 molar ratio), choline chloride: glucose (5:2), choline chloride: 1,2-propanediol (1:3), and citric acid: 1,2-propanediol (1:4) [23,24]. The extraction efficiency for total phenolics and flavonoids was comparable to that of 70% ethanol, though a detailed phytochemical analysis was not conducted. Notably, the citric acid-based extract exhibited strong antibacterial and anti-aging properties, with low toxicity and genotoxicity.

Our study aimed to explore the application of NADESs for extracting the aerial parts of two *Sideritis* taxa, SC and SR, with the ultimate goal of incorporating the extracts into cosmetic products. Five eutectic mixtures were prepared based on the work of Jeong et al. [21], using ingredients such as betaine, glycerol, citric acid, glucose, and sucrose in various proportions. These components are commonly used in cosmetic formulations for skin hydration and conditioning. The total phenolic content (TPC) was determined using the Folin–Ciocalteu method, while the antioxidant potential was assessed using the Ferric Ion Reducing Antioxidant Power (FRAP) and 2,2-diphenyl-1-picrylhydrazyl (DPPH) radical scavenging assays. The results were compared to those of extracts obtained using conventional solvents, specifically aqueous and 70% ethanolic solutions; previous studies have shown the superior extraction efficacy of the latter [9]. The extracts with the highest phenolic content, antioxidant capacity, and favorable physicochemical/organoleptic properties were further analyzed qualitatively and quantitatively with HPLC-MS to identify and quantify their constituents. This study represents the first rigorous phytochemical screening of NADES extracts from two *Sideritis* species exhibiting chemovariability.

## 2. Materials and Methods

### 2.1. Plant Material

The aerial parts of flowering *Sideritis* plants were collected from the Peloponnese, Greece. *S. clandestina* ssp. *peloponnesiaca* was collected from Mountain Helmos in 2014, and *S. raeseri* ssp. *raeseri*, was cultivated in Isoma (Achaia) by ADOLO company and was collected in the summer of 2016. The samples were dried in the shade in a well-ventilated area for 20 days. They were then packed in single-use plastic bags and left in a dark room until analyzed.

### 2.2. Chemicals and Reagents

Methanol (>99.9%) and water used for the HPLC-MS analysis were HPLC-MS grade of Fischer Chemical (Fairlawn, OH, USA). Ethanol (99%) was from Central Chem (Bratislava, Slovakia). Glucose (dextrose anhydrous), acetic acid glacial, and sucrose (D (+) saccharose, laboratory reagent grade) were from Fisher Chemical. Betaine (98% for analysis) was from Acros Organics (Fairlawn, OH, USA), glycerol anhydrous (>99%) from Lachner (Neratovice, Czech Republic), and urea from Chemco by Syndesmos (Athens, Greece). Rutin (purity > 99%) was from Extrasynthese (Genay, France). The Folin–Ciocalteu reagents, sodium acetate trihydrate, and ferrous chloride hexahydrate were produced by Panreac Applichem (Barcelona, Spain). The concentrated hydrochloric acid of analytical grade purity and 2,2-diphenyl-1-picrylhydrazyl (DPPH) were purchased from Sigma-Aldrich (St. Louis, MI, USA). The reagent 2,4,6-tripyridyl-s-triazine (TPTZ) was from Alfa Aesar (Stoughton, MA, USA), the ferrous sulfate heptahydrate from Riedel de Haen (Seelze, Germany), gallic acid (>98%) from Fluka (Buchs, Switzerland), and sodium carbonate from Chem-Lab (Zedelgem, Belgium).

### 2.3. Preparation of NADESs

Five natural deep eutectic solvents developed and characterized in a previous study were evaluated [21]. The preparation followed the heating and stirring method outlined by Dai et al. [19]. All ingredients were added to a beaker and placed on a magnetic heated stirrer. The mixture was stirred for approximately one hour, with temperature measurements taken at regular intervals to maintain a temperature of around 80 °C, until a clear liquid was formed. The amounts of ingredients were calculated based on the mole ratio of each component in the solvent. The names of the solvents and the molar ratios of their ingredients are presented in Table 1.

To facilitate the handling of the eutectic mixtures, which had high viscosity, and to accelerate their conversion into clear liquids, high-purity water was added shortly before the stirring process was completed. This resulted in a final eutectic solvent content of 80% *v*/*v*, according to Jeong et al. [21].

### 2.4. Ultrasound-Assisted Extraction

*S. clandestina* ssp. *peloponnesiaca* and *S. raeseri* ssp. *raeseri* were extracted using the five NADESs, as well as ultrapure water and 70% ethanol. The plant material was chopped in a mixer to increase the surface. After initial experiments, a 200 mg sample of shredded plant material was placed in a 15 mL falcon tube and 10 mL of each solvent was added (1:50 solid-to-solvent ratio). The mixture was extracted in an ultrasonic bath for 90 min, ensuring that the temperature did not exceed 40 °C. After extraction, vacuum filtration was performed using filter paper, and the extracts were collected and stored in the freezer. Each extraction was performed twice.

### 2.5. Pretreatment of Samples for HPLC-MS Analysis

Solid-phase extraction (SPE) was used for the treatment of the two optimal extracts (BGG4 and 70% *v*/*v* ethanol) before HPLC-MS analysis. The extracts were first diluted with ultrapure water in a 1:10 ratio, specifically by adding 1 mL of extract to 9 mL of water. The column used was Strata-X 33 μm Polymeric Reversed Phase (8B-S100-HBJ-S) from Phenomenex (Torrance, CA, USA). The column was activated and equilibrated with 3 mL of high-purity methanol (LC-MS grade), followed by 3 mL of ultrapure water (LC-MS grade). The diluted 10 mL extract was then loaded onto the column and passed through, with pale yellow compounds retained on the solid phase. Afterward, a rinse with 10 mL of water was performed and the compounds were eluted using 6 mL of methanol. The methanolic fraction was vacuum concentrated using a rotary evaporator, followed by complete drying under a high-vacuum pump. The same procedure was applied to the ethanolic extracts to ensure consistent analysis conditions. The final samples were diluted to a concentration of 1.0 and 1.5 mg dry extract/mL in a 70% methanol solvent (LC-MS grade).

### 2.6. HPLC-MS Analysis

The analysis of the extracts was performed by UHPLC-ESI/MS on an instrument 1260 Infinity II of Agilent Technologies Inc. (Santa Clara, CA, USA). Separation was carried out on a Poroshell 120 EC-C18 reverse phase column (120 Å, 2.7 μm, 4.6 × 150 mm), as earlier described [13]. The elution solvents consisted of water with 0.1% acetic acid (A) and methanol (B). In brief, the elution program was as follows: 0 min, 15% B; 8 min, 15% B; 13 min, 35% B; 18 min, 35% B; 19 min, 40% B; 27 min, 40% B; 28 min, 45% B; 35 min, 45% B; 45 min, 75% B; 55 min, 75% B; 59 min, 15% B; 65 min, 15% B. The injection volume was 10 μL, and the flow rate was set to 0.3 mL/min. Rutin at the concentrations of 2, 3, 4, 6, 8, 10, 12, 16, and 20 μg/mL was used as an external standard. The equation was y = 126,832x + 81,400 (R^2^ = 0.9951). Results are expressed as μg rutin equivalents per 100 mg dry extract. The software used to process the chromatograms was OpenLab 3.2.

### 2.7. Determination of Total Phenolics by Folin–Ciocalteu Method

Total phenolic content (TPC) was measured in 96-well plates using the Folin–Ciocalteu reagent [25]. To each well, 18 μL of the sample or standard compound was first added, followed by 36 μL of Folin–Ciocalteu reagent or ddH_2_O (blank) and 144 μL of a 7.5% *w*/*v* Na_2_CO_3_ aqueous solution. After incubation at room temperature for 30 min, absorbance was measured at 620 nm. The TPC was determined based on the standard curve of gallic acid, using the net absorbance of the analytes which is the absorbance of the sample minus the absorbance of the respective blank (sample without Folin–Ciocalteu at the same final volume) and the absorbance of negative control (all reagents with water instead of sample). The concentrations of the standard compound (gallic acid in H_2_O) were 0.05, 0.10, 0.15, 0.20, 0.25, 0.30, and 0.40 mg/mL. The equation was y = 4.2509x + 0.0337 (R^2^ = 0.9962), and the results were expressed as mg of gallic acid equivalents per g of dry weight. Experiments were performed in triplicates for each concentration (at least 5 concentrations per sample) and each experiment was performed at least twice. Averages, standard deviation, and statistical significance were calculated using Microsoft^®^ Excel.

### 2.8. Determination of Antioxidant Activity

The evaluation of antioxidant activity was performed using two different assays: the Ferric-Reducing Antioxidant Power (FRAP) assay and the scavenging of the free radical 2,2-diphenyl-1-picrylhydrazyl (DPPH), following previously established protocols [26,27]. Averages, standard deviation, and statistical significance were calculated using Microsoft^®^ Excel. For every assay, blanks consisting of the sample without the reactant but diluted to the final reaction volume with the appropriate assay solvent were prepared for every concentration and their absorbance values were subtracted from those of the samples.

#### 2.8.1. Ferric Reducing Antioxidant Power (FRAP) Assay

The FRAP solution was prepared by mixing 50 mL of acetate buffer (300 mM, pH 3.6), 10 mL of TPTZ solution (6 mM in 40 mM HCl), and 10 mL of FeCl_3_ solution (20 mM). In the 96-well plate, 55 μL of acetate buffer solution was added to each well, followed by 80 μL of the FRAP solution or ddH_2_O (blank), and finally, 60 μL of the sample or standard compound or sample solvent (negative control). Each analyte was tested in triplicate. Antioxidant capacity was determined using a standard curve of FeSO_4_ [26] using the net absorbance of the samples (original absorbance minus those of the respective blank and negative control). The concentrations of the standard compound (FeSO_4_•7H_2_O in H_2_O) were 0.05, 0.10, 0.15, 0.20, 0.30, and 0.40 mM. The equation used was y = 3.3426x + 0.0097 (R^2^ = 0.9942), and the results were expressed as mmol FeSO_4_ per g of dry weight. The plate was gently stirred and allowed to stand for 5 min in the absence of light. This was followed by measuring absorbance at 595 nm.

#### 2.8.2. DPPH Radical Scavenging Activity (DPPH) Assay

Based on previous protocols [27], five μL of the sample and then 195 μL of the DPPH reagent (0.1 mM in methanol) were added to the wells of a 96-well plate. For the positive control experiments, only methanol and DPPH were used. The measurement was taken after incubating the plate at room temperature for 30 min, in the absence of light, and absorbance was recorded at 540 nm. The results were expressed as % DPPH radical scavenging (%SCAV) from the equation:

%SCAV = [(A_control_ − A_sample_)/A_control_] ∗ 100

where

A_sample_ = net sample absorbance

A_control_ = absorbance of positive control

IC_50_ values, representing the sample concentration required to scavenge 50% of the free DPPH radical, were calculated using GraphPad Prism 7.0 (GraphPad Software, Inc., San Diego, CA, USA). Therefore, smaller IC_50_ values indicate greater antioxidant capacity.

## 3. Results and Discussion

### 3.1. Evaluation of the Extractive Power of the Five Eutectic Solvents and the Antioxidant Properties of the Extracts

#### 3.1.1. Total Phenolic Content

**Figure 1:**
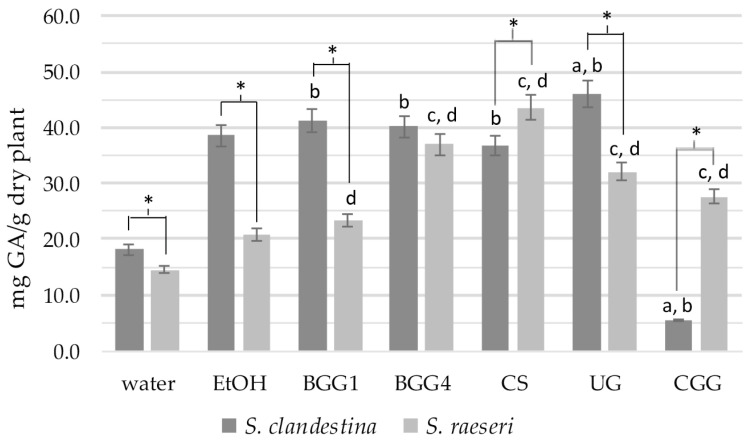
Total phenolic content of the natural deep eutectic extracts of SC and SR. The letters and the asterisks denote statistical significance (*p* < 0.05) as follows: a, vs. SC-EtOH; b. vs. SC-H_2_O; c, vs. SR-EtOH; d, vs. SR-H_2_O; *, vs. the same extract from the other taxon.

Comparing the TPC values of SC extracts, the SC-BGG1, SC-BGG4, and SC-CS extracts did not show statistically significant differences from the SC-EtOH extract (*p* > 0.05) (Figure 1). However, the SC-UG and SC-CGG extracts exhibited statistically significant differences compared to SC-EtOH (with SC-UG showing higher and SC-CGG showing lower values). All extracts except for SC-CGG had a statistically significantly higher phenolic content than the aqueous extract SC-H_2_O (*p* < 0.05). Therefore, we can conclude that the eutectic mixtures BGG1, BGG4, CS, and UG extract phenolic ingredients as effectively as 70% ethanol, demonstrating much higher efficacy than water as an extractive agent. Finally, the eutectic solvent CGG (5.4 ± 1.6 mg GAE/g dry plant) proved to be an inefficient agent for extracting SC phenolic compounds, even compared to water, which had a statistically significantly lower phenolic load (18.1 ± 1.2 mg GAE/g dry plant, *p* < 0.001).

Regarding the TPC of SR extracts, the phenolic content of the eutectic mixtures BGG4, CS, UG, and CGG was statistically significantly higher than that of the 70% ethanolic extracts (Figure 1). Statistical analysis also revealed that the total phenolic content of all eutectic extracts differed significantly from that of the SR-H_2_O extract (*p* < 0.05).

When comparing the extracts of SC and SR with the same solvents, the conventional SC extracts, along with the BGG1 and UG ones, showed statistically significantly higher phenolic content than the respective SR extracts (*p* < 0.05) (Figure 1). The only exceptions were (a) the BGG4 extracts, which did not differ significantly, and (b) the SC-CS and SC-CGG extracts, which had lower phenolic content than the SR extracts.

Overall, TPC analysis revealed significant differences in the phenolic content between the two taxa that are solvent-dependent. In one of our previous studies, however, the *S. raeseri* population exhibited higher TPC than *S. clandestina*, though the plant material and the extraction methods were different from this study [13]. It is well established that factors such as genotype, environmental conditions, season of collection, plant part, and analysis method can influence the extraction, even within the same taxon, let alone when comparing different taxa. Specifically, statistical analysis of the measurements indicates that eutectic solvents are more effective at extracting phenolic components from SR compared to 70% ethanol. In contrast, for SC, all eutectic mixtures—except for the CGG— show extraction capacities similar to that of 70% ethanol. Finally, the CGG solvent does not appear to have a high extractive capacity for phenols in *S. clandestina*, although it performs well in *S. raeseri*.

#### 3.1.2. Ferric Ion Reducing Antioxidant Power

**Figure 2:**
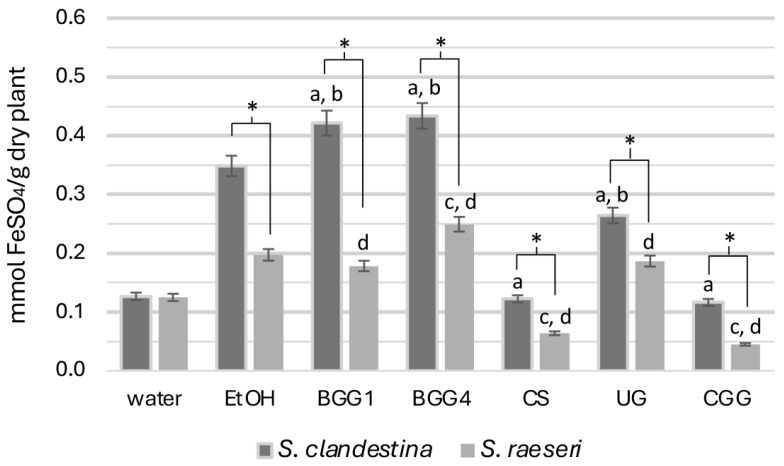
FRAP activity of the natural deep eutectic extracts of SC and SR. The letters and the asterisks denote statistical significance (*p* < 0.05) as follows: a, vs. SC-EtOH; b. vs. SC-H_2_O; c, vs. SR-EtOH; d, vs. SR-H_2_O; *, vs. the same extract from the other taxon.

All SC extracts exhibited significantly higher reducing capacity than SR respective ones, except for the aqueous extracts (SC-H_2_O and SR-H_2_O) which did not show significant differences (*p* > 0.05) (Figure 2).

Specifically, the reducing capacity measurements of the seven SC extracts revealed that betaine-based eutectic mixtures (BGG1, BGG4) exhibited significantly higher antioxidant capacity. In contrast, the SC-CS, SC-UG, and SC-CGG extracts demonstrated statistically significantly lower antioxidant capacity than the 70% ethanolic extract (*p* < 0.05). Additionally, while the reducing capacity of the aqueous extract was generally lower, it was statistically the same as that of SC-CS and SC-CGG. This lower antioxidant capacity for the SC-CS and SC-UG extracts than that of the hydroethanolic extract was unexpected, given their high phenolic load in TPC measurements.

Regarding SR extracts, the SR-BGG1 and SR-UG extracts did not differ significantly from SR-EtOH (*p* > 0.05), indicating similar antioxidant capacity (Figure 2). However, the SR-CS and SR-CGG extracts showed very weak antioxidant activity compared to both SR-EtOH (*p* < 0.001) and SR-H_2_O (*p* < 0.001) extracts. The SR-BGG4 extract, on the other hand, exhibited statistically significantly higher antioxidant capacity than both SR hydroethanolic and aqueous extracts (*p* < 0.05) and was the most effective in this regard. Overall, the betaine-based solvents, especially the BGG4, were the most effective NADESs for extracting reducing compounds.

#### 3.1.3. DPPH Radical Scavenging Activity (DPPH)

Table 2 presents the IC_50_ values of the DPPH radical scavenging activity for each extract. All the NADESs showed very low antioxidant activity, and no matrix effect was observed.

Comparing the DPPH IC_50_ values of extracts with the same solvent between the two taxa, the results showed that the BGG4, CS, and CGG extracts of SC exhibited higher antioxidant capacity compared to the respective extracts of SR, a conclusion consistent with that of the FRAP measurements. Regarding SC, no statistical differences were found in the average IC_50_ values among the different extracts. For SR, the SR-CS and SR-CGG extracts demonstrated a statistically reduced ability to neutralize free radicals compared to the SR-EtOH extract (*p* < 0.05). Additionally, statistical analysis revealed that the SR-UG extract showed significantly higher antioxidant activity than the hydroethanolic extract (*p* < 0.05). Finally, the other eutectic extracts (SR-BGG1, SR-BGG4) and the aqueous extract did not differ statistically from the hydroethanolic extract in terms of IC_50_ values, indicating similar antioxidant capacity.

#### 3.1.4. Selection of the Optimal NADES for Cosmetic Applications

Distinct differences in the extractive power of the eutectic solvents were observed. According to Jeong et al. [21], who developed and studied the NADESs described herein, the pH value of the NADESs significantly influences their extractive power. Specifically, NADESs containing citric acid, betaine, and urea were acidic, neutral, and basic, respectively. However, no clear conclusion can be drawn from our results. Previous studies have focused on the extraction of catechins or anthocyanins [18,21], but the chemical nature of *Sideritis* ingredients differs.

Overall, the betaine-based BGG4, the citric acid-containing CS, and the urea-containing UG performed better than the other solvents in extracting total phenols from both species. However, BGG4 extracts exhibited the highest FRAP values and significantly different radical scavenging activity, particularly when compared to UG (better in SC and worse in SR). Considering that urea can be irritating at concentrations higher than 10% in cosmetic products, as well as its unpleasant organoleptic properties, especially the odor, BGG4 was selected as the optimal solvent. This aligns with the findings of Jeong et al. [21], who additionally evaluated the cost of the raw materials and their suitability for cosmetic products. Regarding glycerol, the most abundant ingredient in the BGG4 solvent, it is important to note that its concentration can be as high as 80% in leave-on products. In addition, the BGG4 was the least viscous and thus easier to handle.

### 3.2. Chemical Composition of BGG4 and EtOH Extracts

HPLC-MS determination of the polar secondary metabolites in the extracts revealed the presence of 24 major compounds, including iridoids (e.g., melittoside), caffeoylquinic acids (e.g., chlorogenic acid), phenylethanoid glycosides (e.g., verbascoside, isoverbascoside, forsythoside A, forsythoside B, and lavandulofolioside), several non-acetylated, mono-acetylated, and diacetylated allosyl glucosyl flavones and their methyl ethers (e.g., hypolaetin, isoscutellarein, luteolin), as well as other flavone derivatives (e.g., apigenin) (Table 3 and Table 4, Figures S1–S4). Identification was performed by comparing the elution times and mass spectra with those of pure standards and with previous studies on *Sideritis* taxa [4,13,28,29,30,31,32,33].

Analysis of *Sideritis* extracts proved that peaks 5, 6, 15, 17, and 19 were found exclusively in SC extracts (Table 3). Quantitative analysis of the metabolites indicated that mono-/diacetylated All-Glc HYP (peaks 20, 21) and diacetylated All-Glc ISC or LUT (peak 22) were quantifiable only in SR, whereas the allosyl methoxy-flavone glycosides (peaks 15, 17 and 19) were detected only in SC (Table 4). Forsythoside A (peak 11) was detected exclusively in BGG4 extracts from both *Sideritis* taxa but was not quantifiable in SR-BGG4.

HPLC-MS analysis of SC extracts showed that both SC-EtOH (11,047.98 μg/100 mg dry extract) and SC-BGG4 (11,093.20 μg/100 mg dry extract) extracts had similar phenolic loads. Specifically, the SC-BGG4 extract contained forsythoside A (peak 11), which was not detected in the SC-EtOH extract. Additionally, the SC-BGG4 extract contained higher amounts of melittoside (peak 2), chlorogenic acid (peak 3), isoverbascoside (peak 12), All-Glc-HYP (peak 10), and All-Glc-HYP-Me (peak 15) compared to SC-EtOH, but lower levels of peak 13, and the unknown compound (peak 4).

When comparing SR extracts, SR-EtOH (8898.09 μg/100 mg dry extract) and SR-BGG4 (8964.98 μg/100 mg dry extract), no significant difference in the overall metabolite content was observed. However, it is important to note that the SR-EtOH extract contained a compound not detected in the SR-BGG4 extract, tentatively identified as acetylated All-Glc-ISC or LUT (peak 14), while forsythoside A (peak 11) was found exclusively in SR-BGG4. In addition, the SR-BGG4 extract exhibited higher levels of melittoside derivative (peak 1), verbascoside (peak 9), All-Glc-HYP (peak 10), isoverbascoside (peak 12), and AcO-All-Glc-ISC or AcO-All-Glc-LUT (peak 18). Finally, the SR-EtOH extract contained higher amounts of chlorogenic acid (peak 3) and (AcO)_2_-All-Glc-HYP (peak 20). The phenylethanoid glycoside (peak 8) was detected in both SR extracts but was not quantifiable in the SR-BGG4 extract.

The SR extracts had a narrower range of metabolites and lower quantities, particularly of iridoids and non-acetylated allosyl flavones, than SC. Our quantitative results of SR metabolites are in agreement with those of Krgovic et al. [9], who determined chlorogenic acid, verbascoside, AcO-All-Glc-ISC, AcO-All-Glc-ISC-Me, AcO-All-Glc-HYP-Me, and (AcO)_2_-All-Glc-HYP-Me in a 70% hydroethanolic extract, e.g., the verbascoside content in their study was about 8.2 mg/g vs. 11.1 mg/g in our study; the only notable difference is the higher chlorogenic acid content in our study (1.9 vs. 14.9 mg/g, respectively). The quantitative comparison of SR and SC herein differs from that of our previous work [13], but that study was focused on metabolomic comparisons and used a different extraction strategy and smaller amounts of plant material; in addition, the plant material of SR was different. Herein, we conducted larger-scale extractions, and our purpose was to compare green extraction strategies and evaluate the potential for cosmetic applications. Even though there is space for meticulous optimization of the extraction processes, we demonstrate that the extractive capacity of BGG4 was comparable to that of 70% EtOH in both cases (SC and SR) and offer a new extraction alternative.

The most abundant compounds in all SC and SR green extracts were chlorogenic acid and verbascoside. Both are valuable ingredients for cosmetic products since they have strong antioxidant, anti-inflammatory, photoprotective, and favorable extracellular matrix-modulating activities [34,35,36]. Concerning the unusual *Sideritis* flavones which are in great amounts, there is an accumulation of evidence about their potential skin-care roles. The flavones with an uncommon 8-hydroxyl moiety, i.e., isoscutellarein, 4′-*O*-methylhypolaetin, and 4′-*O*-methylisoscutellarein, have been shown to possess strong antioxidant activity, dose-dependent inhibitory activity of collagenase and advanced glycation end-product formation, anti-allergenic activity and the latter compound inhibits UVB-induced Matrix Metallo Proteinase-1 expression inhibition [37]. In addition, apigenin 7-*O*-p-coumaroyl-glucosides and the 4′-methyl-hypolaetin 7-*O*-[6‴-O-acetyl-β-d-allopyranosyl]-(1→2)-β-d-glucopyranoside have anti-hyaluronidase activity [7]. The fact that their occurrence is limited has hindered comprehensive bioactivity studies; however, growing evidence on their constituents and extracts highlights their potential as effective skincare ingredients.

## 4. Conclusions

This study systematically evaluated the extraction efficiency of five different natural deep eutectic solvents (NADESs) for extracting bioactive compounds from *Sideritis* species, i.e., *Sideritis clandestina* ssp. *Peloponnesiaca*, and *Sideritis raeseri* ssp. *raeseri*. Unlike conventional solvents which are commonly used for plant extraction, this research explores the potential of NADESs—specifically mixtures of betaine, glycerol, glucose, urea, citric acid, and sucrose—as more sustainable and efficient alternatives. Our findings highlight the distinct advantages of betaine-based (BGG4), citric acid-based (CS), and urea-based (UG) NADESs in comparison to conventional solvents like ethanol, particularly in terms of total phenolic content, antioxidant activity, and radical scavenging ability. Among these, BGG4 proved to be the most effective solvent, offering superior extraction efficiency and significantly higher FRAP values and DPPH scavenging activity, especially in SC. Furthermore, BGG4’s lower viscosity, cost-effectiveness, and suitability for cosmetic applications, as demonstrated by Jeong et al. [21], make it a promising candidate for incorporation into cosmetic formulations. However, variations in the extraction of the major SC and SR metabolites with BGG4 were observed depending on the taxon, highlighting the complexity of plant chemistry and its interactions with solvents. Notably, the identification of high levels of skin-beneficial secondary metabolites, such as chlorogenic acid, verbascoside, and 8-hydroxyflavone derivatives, in ironwort extracts underscores the value of these extracts in cosmetic and dermatological formulations. This finding also highlights a new opportunity for the valorization of mountain tea and emphasizes the need for efforts to protect and cultivate the local endemic *Sideritis* species. This study provides valuable insights into the use of natural deep eutectic solvents (NADEs) for the extraction of bioactive compounds from *Sideritis* species. These eco-friendly alternatives to traditional solvents offer a sustainable approach to developing natural cosmetic products, aligning with the increasing demand for environmentally conscious formulations.

## Figures and Tables

**Table 1:** Natural deep eutectic solvents and molar proportions of their ingredients.

NADESs	Ingredients (Molar Ratio)
BGG1	Betaine–glycerol–glucose (4:5:1)
BGG4	Betaine–glycerol–glycose (4:20:1)
UG	Urea–glycerol (1:1)
CS	Citric acid–sucrose (1:1)
CGG	Citric acid–glycerol–glucose (1:2:1)

**Table 2:** Determination of the DPPH radical scavenging activity of the SC and SR extracts. The results are expressed as IC_50_ values and as mean value ± standard deviation.

	DPPH (IC_50_ mg Initial Dry Plant/mL)	
Extract	*Sideritis clandestina* ssp. *peloponnesiaca*	*Sideritis raeseri* ssp. *raeseri*
BGG1	4.163 ± 0.771	5.425 ± 0.293
BGG4	3.811 ± 0.135 *	5.962 ± 0.784 *
CS	5.555 ± 0.890 *	7.685 ± 0.868 ^c,d,^*
UG	4.859 ± 0.330	4.174 ± 0.487 ^c^
CGG	4.543 ± 0.351 *	7.357 ± 0.797 ^c,^*
EtOH	4.218 ± 0.420	5.584 ± 0.548
H_2_O	4.330 ± 0.280	5.399 ± 0.762

Note. Superscripts denote the statistical significance (*p* < 0.05) as follows: c, vs. SR-EtOH; d, vs. SR-H_2_O; *, vs. the same extract from the other taxon.

**Table 3:** List of polar metabolites in SC and SR ethanol and BGG4 extracts. The HPLC-MS elution times, the molecular ion from the negative ionization, and other molecular adduct ions from positive and negative ionization as well as references to previous studies.

	tR (min)	Tentative Identification	Molecular Ion [M−H]^−^ (*m*/*z*)	Other Ions from Both Negative and Positive Ionization (*m*/*z*)	Occurrence	Reference
1	3.39	Μelittoside derivative	523	583 (M+Hac−H)^−^/1047 (2M−H)^−^	all	[4]
2	6.49	Melittoside *	523	583 (M+Hac−H)^−^/1047 (2M−H)^−^	all	[13]
3	17.83	3-Caffeoylquinic acid (chlorogenic acid) *	353	707 (2Μ−H)^−^/377 (M+Na)^+^/731 (2M+Na)^+^	all	
4	20.14	Unknown	669	1339 (2M−H)^−^/355 (M+H+K)^2+^/693 (M+Na)^+^/1363 (2M+Na)^+^	all	
5	23.90	7-*O*-Acetyl-8-epiloganic acid	417	441 (M+Na)^+^	SC-EtOH, SC-BGG4	[13]
6	24.82	Ajugoside *	389	449 (M+Hac−H)^−^/413 (M+Na)^+^	SC-EtOH, SC-BGG4	[13]
7	26.83	Unknown	785	392 (M−2H)^−^/809 (M+Na)^+^/413 (M+H+K)^2+^	all	
8	27.20	Forsythoside B or Lavandulofolioside	755	377 (M−2H)^2−^/779 (M+Na)^+^/398 (M+H+K)^2+^	all	[28]
9	28.10	Verbascoside *	623	1247 (2M−H)^−^/647 (M+Na)^+^/1271 (2M+Na)^+^	all	[13]
10	29.08	All-Glc-HYP	625	1251 (2M−H)^−^/649 (M+Na)^+^	all	[30]
11	33.04	Forsythoside A	623	647 (M+Na)^+^	SC-BGG4, SR-BGG4	[30]
12	33.67	Isoverbascoside *	623	645 (M+Na−2H)^−^/311 (M−2H)^−^/647 (M+Na)^+^	all	[13]
13	34.03	Leucosceptoside isomer//AcO-All-Glc-ISC or AcO-All-Glc-LUT	667 (M_1_)// 651 (M_2_)	1335 (2M_1_−H)^−^/691 (M_1_+Na)^+^// 653 (M_2_+H)^+^/675 (M_2_+Na)^+^	M_1_: in SC-EtOH, SC-BGG4, M_2_: all	[31,32]
14	34.67	AcO-All-Glc-HYP// AcO-All-Glc-ISC or AcO-All-Glc-LUT	667 (M_1_)// 651 (M_2_)	1335 (2M_1_−H)^−^/691 (M_1_+Na)^+^// 653 (M_2_+H)^+^/675 (M_2_+Na)^+^	M_1_: all, M_2_: SC-BGG4, SR-EtOH	[32]
15	35.71	All-Glc-HYP-Me	639	1279 (2M−H)^−^/663 (M+Na)^+^/1303 (2M+Na)^+^	SC-EtOH, SC-BGG4	[32]
16	36.35	AcO-All-Glc-HYP	667	1335 (2M−H)^−^/691 (M+Na)^+^	all	[32]
17	40.70	AcO-All-Glc-ISC-Me or AcO-All-Glc-LUT-Me or AcO-All-Glc-Chrys// AcO-All-Glc-HYP-Me isomer	665 (Μ_1_)// 681 (Μ_2_)	683 (M_2_+H)^+^	SC-EtOH, SC-BGG4	[29,30]
18	42.58	AcO-All-Glc-ISC or AcO-All-Glc-LUT	651	1303 (2M−H)^−^/675 (M+Na)^+^/1327 (2M+Na)^+^	all	[29,30]
19	43.31	AcO-All-Glc-HYP-Me isomer	681	1363 (2M−H)^−^	SC-EtOH, SC-BGG4	[30]
20	43.98	(AcO)_2_-All-Glc-HYP	709		all	[29,30]
21	44.66	AcO-All-Glc-HYP	667	669 (Μ+H)^+^	all	[29,30]
22	46.40	(AcO)_2_-All-Glc-HYP-Me// (AcO)_2_-All-Glc-ISC or (AcO)_2_-All-Glc-LUT	723 (Μ_1_)// 693 (Μ_2_)	747 (M_1_+Na)^+^//695 (Μ_2_+H)^+^	M_1_: in SC-EtOH, SC-BGG4 M_2_: all	[29,30]
23	47.79	(AcO)_2_-All-Glc-HYP	709	1419 (2M−H)^−^/733 (M+Na)^+^/1443 (2M+Na)^+^	all	[29]
24	49.31	Apigenin coumaroylrutinoside//(AcO)_2_-All-Glc-ISC or (AcO)_2_-All-Glc-LUT	723 (Μ_1_)// 693 (Μ_2_)	725 (Μ_1_+H)^+^/747 (M_1_+Na)^+^//695 (Μ_2_+H)^+^	M_1_: in SC-EtOH M_2_: all	[29,33]

*: confirmed with pure standard compounds. Abbreviations: All: allosyl, Glc: glucosyl, AcO: acetoxy, Me: methyl, API: apigenin, HYP: hypolaetin, Chrys: chrysoeriol, ISC: isoscutellarein, LUT: luteolin.

**Table 4:** List of polar metabolites and their concentrations (μg rutin equivalents/100 mg of dry extract) in SC and SR 70% ethanol and BGG4 extracts.

Peak Number	Compound	Category	SC-EtOH	SC-BGG4	SR-EtOH	SR-BGG4
1	Melittoside derivative	Iridoid	387.35 ± 10.64 *	387.79 ± 46.25	261.57 ± 12.30 *	463.81 ± 64.08 ^c^
2	Melittoside	Iridoid	433.31 ± 16.34 *	665.11 ± 74.68 ^a,^*	244.15 ± 1.71 *	197.29 ± 20.99 ^c,^*
3	3-caffeoylquinic acid (chlorogenic acid)	Hydroxycinnamic acid derivative	1535.73 ± 61.37	1779.73 ± 102.13 ^a,^*	1490.67 ± 59.24	1068.76 ± 19.09 ^c,^*
4	Unknown		842.11 ± 46.14 *	394.93 ± 26.97 ^a,^*	384.85 ± 52.10 *	333.45 ± 36.05 *
7	Unknown		555.46 ± 82.08	444.74 ± 1.82	373.51 ± 50.48	n.q.
8	Forsythoside B or Lavandulofolioside	Phenylethanoid glycoside	611.09 ± 20.76 *	555.15 ± 48.18	215.12 ± 21.22 *	n.q.
9	Verbascoside	Phenylethanoid glycoside	1482.82 ± 19.94 *	1545.71 ± 45.55 ^a,^*	1109.75 ± 94.96 *	1363.12 ± 9.81 ^c,^*
10	All-Glc-HYP	Non-acetylated allosyl flavone	345.73 ± 35.33 *	420.61 ± 6.86 ^a,^*	554.44 ± 45.45 *	783.57 ± 21.53 ^c,^*
11	Forsythoside A	Phenylethanoid glycoside	-	341.13 ± 23.49	-	n.q.
12	Isoverbascoside	Phenylethanoid glycoside	n.q.	245.78 ± 1.47 *	360.71 ± 27.86	498.41 ± 61.85 ^c,^*
13	Leucosceptoside isomer// AcO-All-Glc-ISC or AcO-All-Glc-LUT	Phenylethanoid glycoside// Acetylated allosyl flavone glucoside	781.93 ± 44.17	532.40 ± 46.32 ^a,^*	763.80 ± 67.28	945.71 ± 11.46 ^c,^*
14	AcO-All-Glc-HYP//AcO-All-Glc-ISC or AcO-All-Glc-LUT	Acetylated allosyl flavone glucoside	305.57 ± 18.92	239.04 ± 13.70	233.17 ± 43.43	-//n.q.
15	All-Glc-HYP-Me	Allosyl flavone glucoside	117.96 ± 3.48	247.56 ± 35.35 ^a^	-	-
16	AcO-All-Glc-HYP	Acetylated allosyl flavone glucoside	651.43 ± 55.64 *	764.20 ± 58.54 ^a,^*	931.50 ± 108.68 *	980.92 ± 31.42 *
17	AcO-All-Glc-ISC-Me or AcO-All-Glc-LUT-Me or AcO-All-Glc-Chrys//AcO-All-Glc-HYP-Me isomer	Acetylated allosyl flavone glucoside	536.62 ± 45.19	423.64 ± 51.21	-	-
18	AcO-All-Glc-ISC or AcO-All-Glc-LUT	Acetylated allosyl flavone glucoside	971.94 ± 3.00 *	956.60 ± 111.21	150.72 ± 33.87 *	747.30 ± 26.04 ^c,^ *
19	AcO-All-Glc-HYP-Me isomer	Acetylated allosyl flavone glucoside	597.30 ± 25.51	619.09 ± 10.25	-	-
20	(AcO)_2_-All-Glc-HYP	Acetylated allosyl flavone glucoside	n.q.	n.q.	853.93 ± 93.95	228.07 ± 22.19 ^c^
21	AcO-All-Glc-HYP	Acetylated allosyl flavone glucoside	n.q.	n.q.	183.79 ± 66.40	309.96 ± 8.21 ^c^
22	[(AcO)_2_-All-Glc-ISC] or [(AcO)_2_-All-Glc-LUT]	Acetylated allosyl flavone glucoside	n.q.	n.q.	315.18 ± 4.40	339.99 ± 19.12
23	(AcO)_2_-All-Glc-HYP	Acetylated allosyl flavone glucoside	295.65 ± 42.56 *	295.23 ± 5.54 *	500.21 ± 69.20 *	491.29 ± 69.24 *
24	Apigenin coumaroylrutinoside//(AcO)_2_-All-Glc-ISC or (AcO)_2_-All-Glc-LUT	Other flavones//Acetylated allosyl flavone glucoside	210.14 ± 6.28	234.76 ± 5.13 ^a^	215.17 ± 32.32	213.33 ± 20.43

Abbreviations: All: allosyl, Glc: glucosyl, AcO: acetoxy, Me: methyl, API: apigenin, HYP: hypolaetin, Chrys: chrysoeriol, ISC: isoscutellarein, LUT: luteolin, n.q.: not quantifiable. Values represent the average ± standard deviation of measurements of two samples per extract twice. Superscripts denote the statistical significance (*p* < 0.05) as follows: a, vs. SC-EtOH; c, vs. SR-EtOH; *, vs. the same extract from the other taxon.

## Data Availability

The original contributions presented in this study are included in the article’s material and the Supplementary Information. Further inquiries can be directed to the corresponding author.

## Supplementary Materials

The following supporting information can be downloaded at: https://www.mdpi.com/article/10.3390/antiox14010068/s1, Figure S1: Representative Total Ion Chromatogram of SC-EtOH (1 mg/mL) under positive (upper panel) and negative ESI ionization (lower panel) modes. Figure S2: Representative Total Ion Chromatogram of SC-BGG4 (1 mg/mL) under positive (upper panel) and negative ESI ionization (lower panel) modes. Figure S3: Representative Total Ion Chromatogram of SR-EtOH (1 mg/mL) under positive (upper panel) and negative ESI ionization (lower panel) modes. Figure S4: Representative Total Ion Chromatogram of SR-BGG4 (1 mg/mL) under positive (upper panel) and negative ESI ionization (lower panel) modes.
