# Supplementary material for: Natural Deep Eutectic Solvents as Green Alternatives for Extracting Bioactive Compounds from Sideritis Taxa with Potential Cosmetic Applications
Source: Antioxidants (Basel). 2025 Jan 9;14(1):68. doi: 10.3390/antiox14010068 (PMC11759769; doi:10.3390/antiox14010068)
Supplement: Supplementary file 1 [file antioxidants-14-00068-s001.zip › antioxidants-3387154-supplementary.pdf]

SUPPLEMENTARY INFORMATION

Natural Deep Eutectic Solvents as Green Alternatives for Extracting Bioactive Compounds from *Sideritis* Taxa with Potential Cosmetic Applications

Lamprini Zissi, Virginia D. Dimaki, Vassiliki S. Birba, Vassiliki C. Galani, Vassiliki Magafa, Sophia Hatziantoniou, Fotini N. Lamari*

Department of Pharmacy, School of Health Sciences, University of Patras, 26504 Patras, Greece
*Correspondence: flam@upatras.gr; Tel.: +30 2610962335

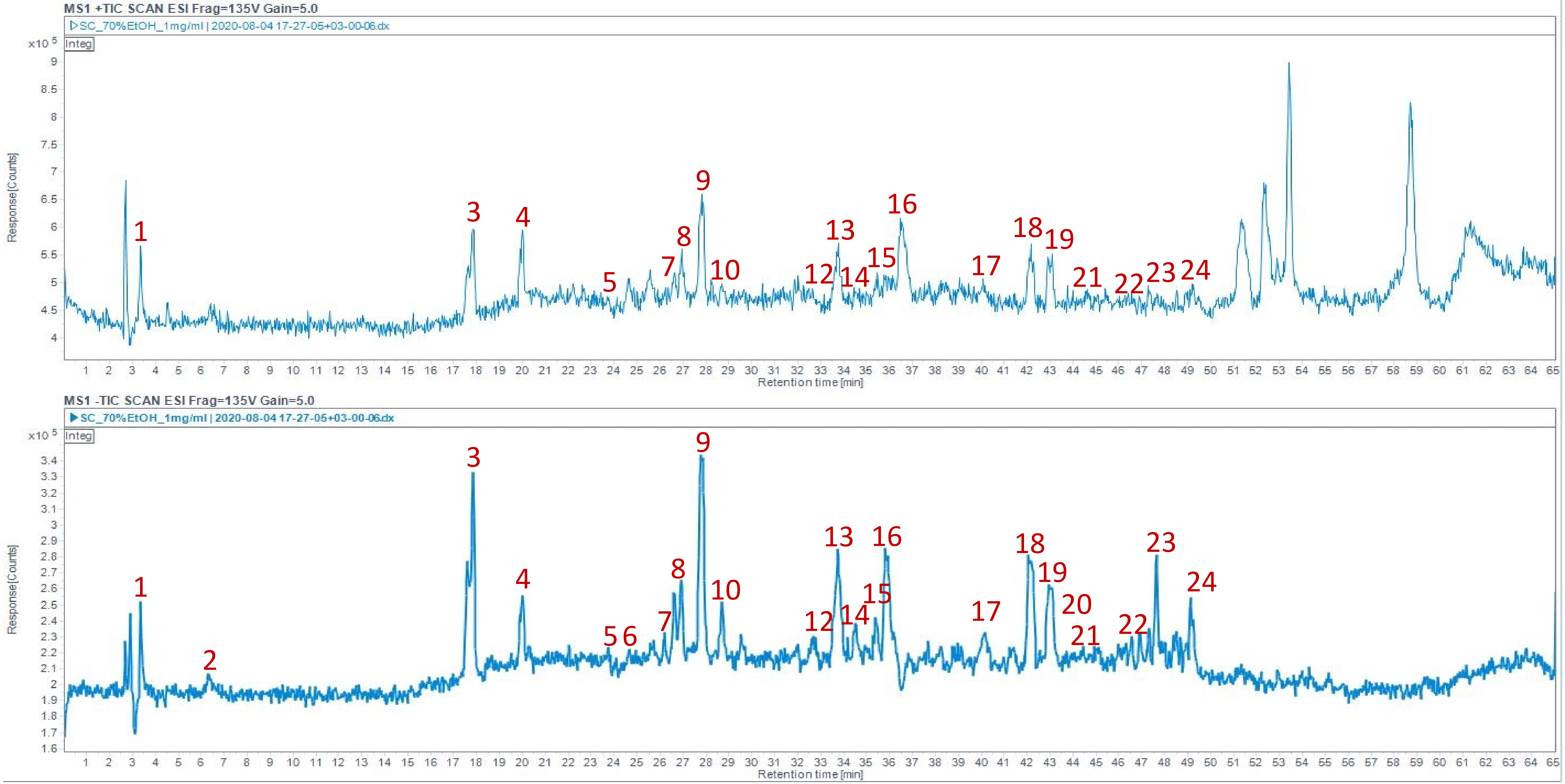

Figure S1. Representative Total Ion Chromatogram of SC-EtOH (1 mg/mL) under positive (upper panel) and negative ESI ionization (lower panel) modes.

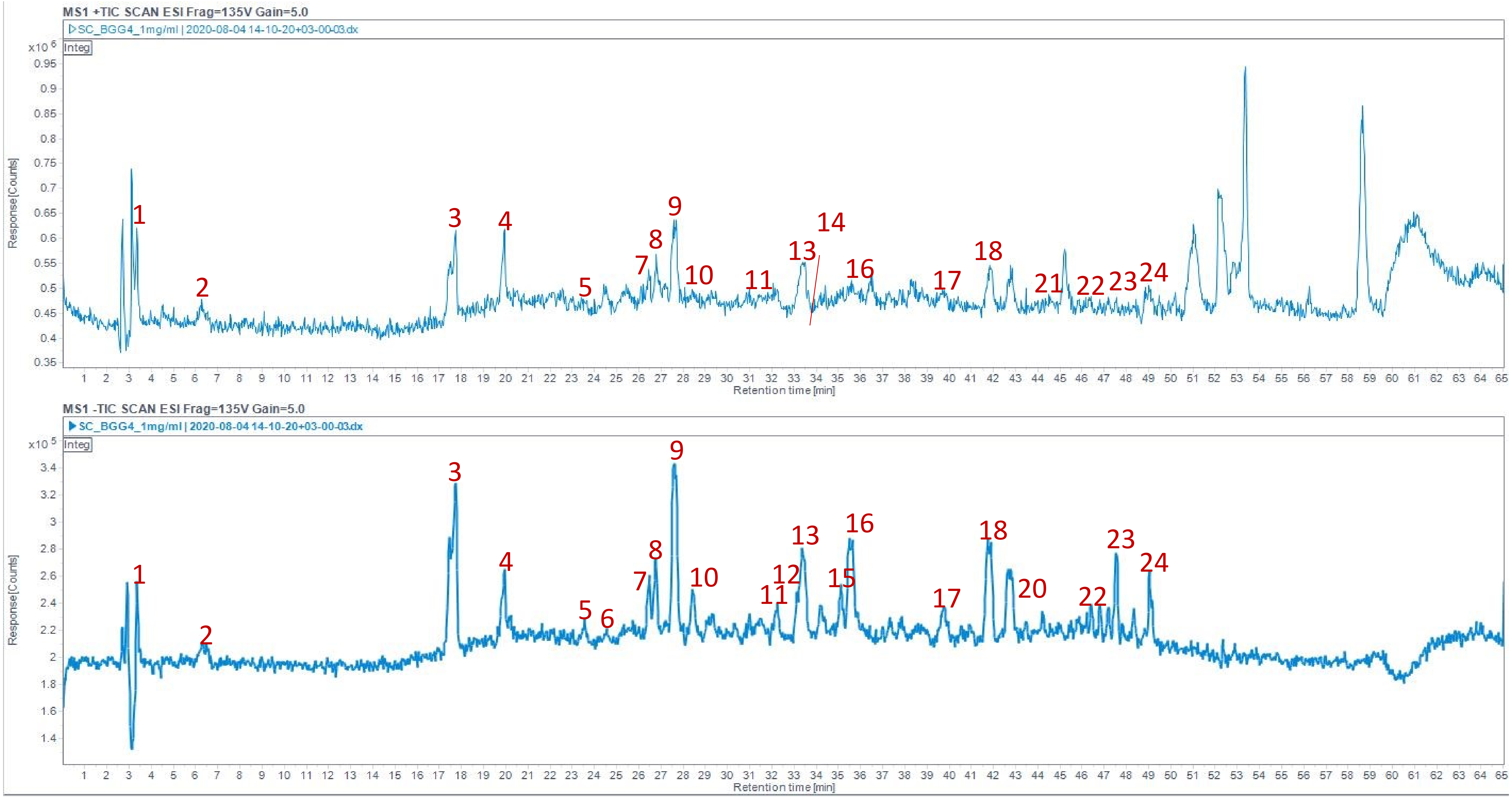

Figure S2. Representative Total Ion Chromatogram of SC-BGG4 (1 mg/mL) under positive (upper panel) and negative ESI ionization (lower panel) modes.

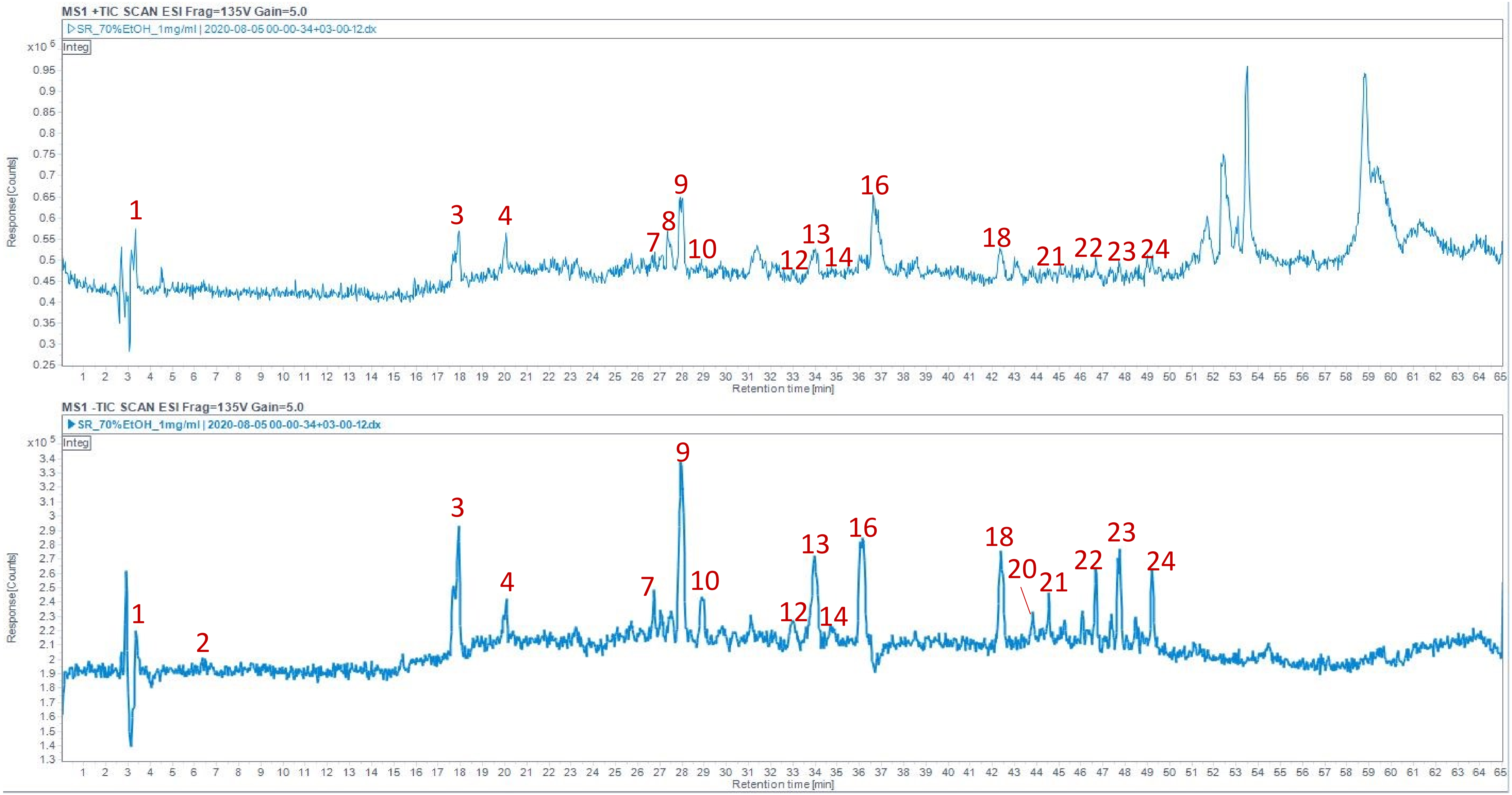

Figure S3. Representative Total Ion Chromatogram of SR-EtOH (1 mg/mL) under positive (upper panel) and negative ESI ionization (lower panel) modes.

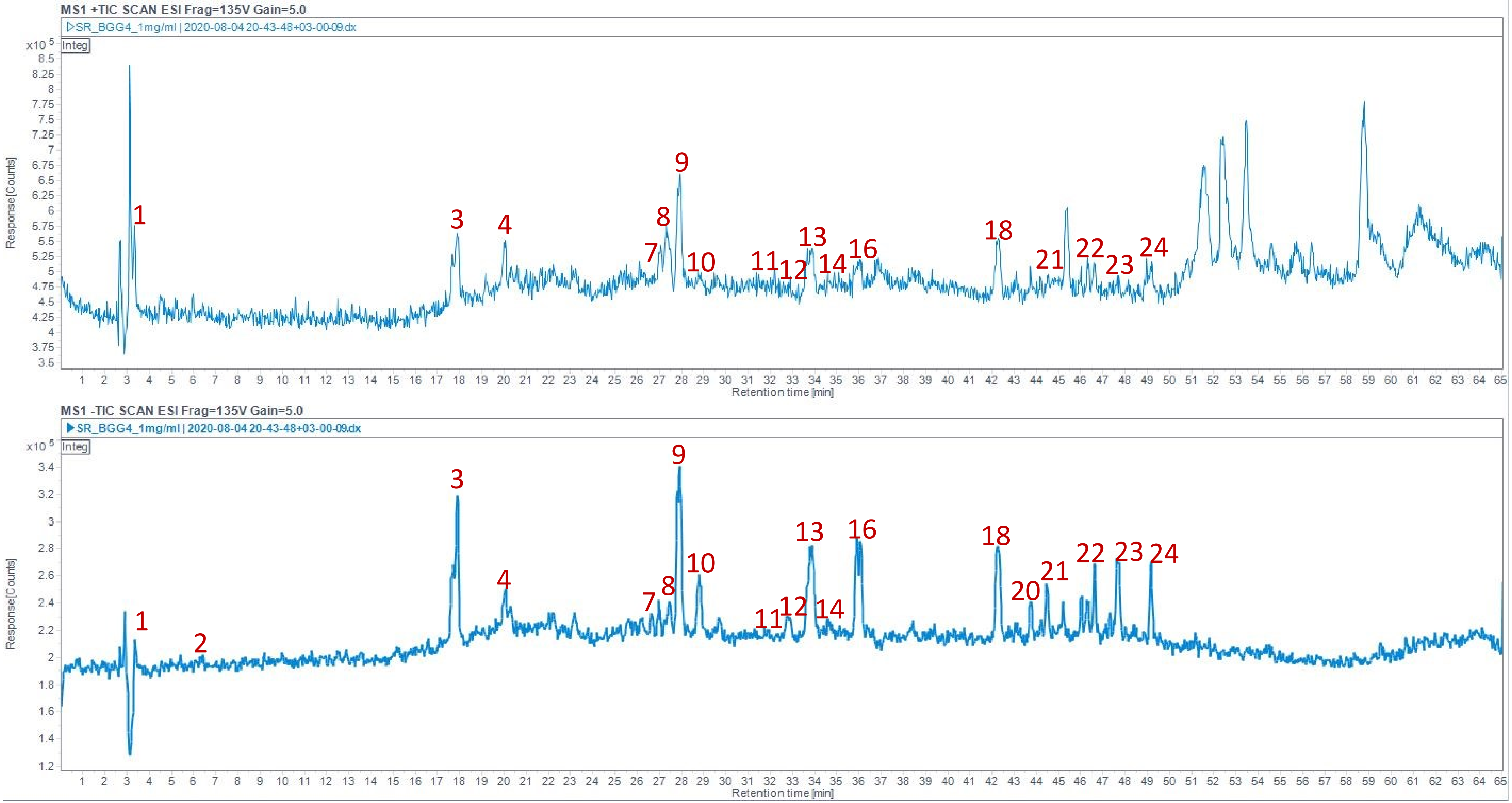

Figure S4. Representative Total Ion Chromatogram of SR-BGG4 (1 mg/mL) under positive (upper panel) and negative ESI ionization (lower panel) modes.
